# Is a Consumer Perception of Salt Modification a Sensory or a Behavioural Phenomenon? Insights from a Bread Study

**DOI:** 10.3390/foods9091172

**Published:** 2020-08-25

**Authors:** Aleš Kuhar, Mojca Korošec, Anja Bolha, Igor Pravst, Hristo Hristov

**Affiliations:** 1 Biotechnical Faculty, University of Ljubljana, Jamnikarjeva 101, SI-1000 Ljubljana, Slovenia; mojca.korosec@bf.uni-lj.si (M.K.); anja.bolha@bf.uni-lj.si (A.B.); 2 Nutrition Institute, Tržaška cesta 40, SI-1000 Ljubljana, Slovenia; igor.pravst@nutris.org (I.P.); hristo.hristov@nutris.org (H.H.)

**Keywords:** bread, salt reduction, intensity perception, hedonic liking, latent class clustering, PCA

## Abstract

Salt plays a major role in food manufacturing and affects the technological and sensory properties of foods. At the same time, high dietary salt intake increases the risk of cardiovascular diseases and represents a considerable public health concern. In many populations, bread is a major contributor to salt intake and is therefore targeted by health policies recommending salt reduction reformulations. However, producers are often reluctant to reduce salt content due to fears of potential negative effects on consumer acceptability and drops in sales. The present work aims to assess the effect of salt content on consumers’ hedonic liking and perception of saltiness intensity, as well as the interaction of these two. The study was conducted using two market-leading bread types (white and multigrain) and bread samples with the national average (reference), reduced (−15%) and increased (+10%) salt levels. A sensory evaluation study (*n* = 200) was done including a questionnaire on attitudes and behaviour regarding bread and salt, enabling the exploration of individual differences in reactions to modified levels of salt content. The latter only affected hedonic liking for the multigrain sample with 15% salt reduction but not for others, which discloses the importance of the bread matrix; and it did not affect the perception of saltiness intensity. Penalty analysis revealed that perceived non-optimal saltiness results in significantly penalised hedonic liking scores. Segmentation based on perceived saltiness disclosed the explanatory importance of underlying consumer behaviour dimensions which should be considered in designing bread reformulations.

## 1. Introduction

Dietary habits and lifestyle affect quality of life and the sustainability of health systems. Unhealthy food choices and a lack of physical activity contribute significantly to the burden of chronic non-communicable diseases (NCDs) and to the costs of health care funds [1]. Among NCDs, cardiovascular diseases (CVDs) are considered the number one cause of death (over 30% of all global deaths) and many of these deaths are preventable, inter alia with suitable modifications to unhealthy diets [2]. High sodium intake is considered as the most important dietary factor related to the global burden of CVDs [3,4]; globally, over half of diet-related deaths and about two-thirds of diet-related disability-adjusted life-years (DALYs) are due to high sodium intake [2]. A direct relationship has long been established between hypertension and sodium intake [2,3,5,6] and a notable proportion of deaths due to CVDs can be attributed to high blood pressure. Since even a very small reduction in blood pressure can have large positive effects on systolic blood pressure [7,8,9], a significant decrease in mortality rate can be achieved with a reduction of dietary salt intake, making this an important public health intervention in the CVDs domain. The World Health Organisation (WHO) has set the population target for salt intake at an equivalent of 5 g of salt daily and a 30% reduction in mean population salt intake by 2025 [3,10], and a number of activities have been implemented by healthcare authorities to reach them. However, in many countries, salt intake is still well above this target, commonly even double the recommended value [11,12,13,14].

In addition to improving the scientific understanding of the negative consequences of excessive salt consumption and appropriate consumer information and awareness, the modification of food products, either stimulated by consumer demand for healthier products or supported by government incentives, has been identified as the key pillar of salt intake reduction [15]. This approach is particularly promising in populations where the majority of dietary salt is consumed via food products such as bread, meat products, cheese, savoury snacks, sauces and condiments, and ready meals, which are recognized as the major contributors to salt intake [11]. The great majority of dietary salt consumed in developed countries originates from processed food, while only 15% is added during cooking or at the table, and 5% is naturally present in foods [16]. Therefore, a focus on reducing the amount of salt added by food producers is well-justified. Some governments already work with industry to reduce the salt content of foods and some progress has been made [17,18], but there remains a pressing need for further improvement.

Focusing on a specific food product group, reducing the level of salt in breads and bakery products would have a major impact on global public health due to both the quantity consumed and their salt content [11,19,20]. Indeed, salt in bread is a critical ingredient from the perspective of production processes and technology (e.g., yeast activity, gluten network formation, bread volume, dough handling, shelf life, etc.) as well as a sensory characteristic [21,22,23]. Therefore, the bakery industry and other food manufacturers are often reluctant to reduce the salt just because of public health targets since they face various challenges when reformulating food products. The reformulated products have to achieve satisfactory sensory quality while maintaining safety, shelf life, and commercial viability [24]. In order to preserve the basic properties of the food and prevent negative consequences on product acceptability and a drop in sales, food product reformulation must be undertaken in a consumer-driven manner [15].

Studies show that a considerable proportion of the population is neither aware of salt intake recommendations nor interested in salt reduction [25], and this limits the consumer demand-side stimulation for food product reformulation. Furthermore, some studies have shown that the use of “low salt” nutrition claims alone (without sensory evaluation) can have a negative impact on purchasing decisions [26]. A special challenge for the food industry is therefore to successfully apply the “stealth reformulation approach” [15], where the consumer will not receive any information on salt reduction, nor perceive the difference in taste, while preserving the food’s acceptability and technological properties [22,27]. Based on reported studies, gradual silent reformulation that reduces salt in bread by 20–30% without the use of substitutes may be advisable [28,29,30,31,32]. Further, several studies report that stealthy salt reduction in breads had no sales impacts [27,33,34]. When using (for example) sourdough as a taste enhancer, further reductions of up to 50% may be achievable [35,36].

Slovenia is an example of a country with a very high daily salt intake (about 12 g daily, with over 5 g derived from purchased food [37]), with bread and bakery products being the key contributors [38]. Average yearly per capita in-home consumption of bread was almost 27 kg in 2018 [39], while mean salt content in plain bread has remained unchanged for a decade at around 1.3 g/100 g [40,41]. This is despite the efforts envisaged in the national action plan [42] which anticipated a very ambitious reduction of the salt content in bread to 1.0 g per 100 g by 2020.

The barriers to achieving the objectives of the national salt reduction plan are fears regarding consumer acceptance of reformulated breads and a consequential loss of sales. Readiness to reformulate was high with the largest national bakeries, but the smaller bakeries were not interested. As the former feared the “first mover risk” and loss of consumers to the producers of non-reformulated breads, the notion failed to get traction. The largest producers also claimed they already produced low salt breads, but that their market shares remained marginal [43].

In 2019, the National Association of Millers and Bakers established a salt-reduction taskforce of their own initiative, which committed to preparing a factual basis for reducing salt levels in their products that also included the provision of relevant guidelines on the underlying technology and consumer response.

Considering the abovementioned challenges, the objective of this study was to explore individual differences between consumer reactions to reductions in the salt content of the leading breads in the Slovenian market. Also, the importance of the bread matrix was investigated since the best-selling white and multigrain breads were considered for reformulation. The results provide valuable insights that can support national bread reformulation activities and the managerial decisions of individual bakeries in this regard.

## 2. Materials and Methods

### 2.1. Bread Sample Formulations

The tested bread formulations were based on the average salt content of white and multigrain breads with the highest market shares on the Slovenian market. The reference salt content was determined with the support of members of the National Association of Millers and Bakers, who reported the individual salt content for the best-selling bread stock keeping units (SKUs), which cumulatively represented approximately 50% of the total production of each participating bakery in the category of white breads and standard multigrain breads (wheat + rye). In total, 6 of the largest bakeries provided 47 SKUs of white bread, where the average salt content was 1.32 ± 0.13 g/100 g and 38 SKUs of multigrain bread, for which the average salt content was 1.26 ± 0.10 g/100 g. These concentrations are in line with previous independent reports [40,41]. The reference saltiness level for both bread types was therefore set at 1.3 g/100 g of bread and the samples were produced using the recipes in Table 1.

A combination of scientific and practical considerations were considered for determining the salt level modification in the testing samples following the industrial partners’ interests not to exceed the reduction range past the point when the acceptability clearly decreases. For the low-salt bread formulation, the salt content was reduced by 15% to 1.1 g of salt per 100 g of bread following the recommendation of the national plan on salt reduction [42], while the high salt bread formulation was set to 1.4 g of salt, implying the national market reality. For the white bread (WB) samples, 100% commercial all-purpose wheat flour (type 500) was used with 2.5% fresh baker’s yeast, 60% tap water and 2.5% general bread improver for regulating flour quality, bread consistency and quality of the dough (GLUTOPAN^®^; Lesaffre, Marcq-en-Baroeul, France). For the multigrain bread (MGB) samples, a mixture of 70% commercial all-purpose wheat flour (type 500) and 30% rye flour (type 1250) was used, with 2.5% yeast, 60% tap water and 2.5% general bread improver (GLUTOPAN^®^).

The quantity of added salt differed by type of bread due to differences in the water absorption and consequently water content in the bread [44,45,46].

Bread samples were produced in a test laboratory of an industrial partner (one of the leading national bakeries) in a single batch for each of the six formulations. After baking and cooling, the loaves were individually packed and frozen. The required quantity of samples was taken from the refrigerator for defrosting each evening before consumer tests. On the morning of the test day, samples were cut into 50 g portions (10 mm slices) containing both the crust and the core and stored in a plastic bag to maintain freshness during the test. The samples were numbered with an assigned three-digit random code.

### 2.2. Consumer Testing

The sensory analysis was conducted using an untrained sample of Slovenian bread consumers, applying the principles of convenience sampling. The targeted size of the total sample was 200 participants. Testing was conducted in four shopping malls located in various geographical regions of Slovenia (Central, North-East, West and South), where, upon agreement with the management of the premise, an isolated testing area was set up ensuring conditions conforming to the strict requirements of hedonic sensory testing. The trial subjects were recruited from among the shopping mall visitors using a non-random selection mechanism (non-probability sampling) until the desired number of completed tests was obtained for each gender and age cohort. The participants were chosen only if they positively responded that they: (1) regularly consume bread (more than once a week), (2) have no known food allergies or intolerances (e.g., wheat, gluten) and (3) have no abnormalities related to smell and taste.

Upon their arrival to the testing area, which accommodated a maximum of 3 consumers at a time, trained researchers invited the positively screened subjects to a mobile testing booth. Prior to the test, survey assistants explained the protocol, whereas the objectives of the study (i.e., salt reduction in breads) were not explicitly revealed. The first sample of bread given to the respondents was intended for them to get acquainted with the principles of consumer sensory evaluation and the survey protocol. They were instructed on how to rate their degree of liking/disliking and the perception of saltiness intensity. The order in which they were presented with samples was randomised to prevent “carry-over” and “first order” effects [47]. Samples were presented on a white paper plate and assessors were served water and encouraged to cleanse their palate between samples. Each sample was presented with its corresponding three-digit random code. Participants evaluated white breads (WB) first, followed by multi-grain breads (MGB) in two separate sessions. They were first asked to rate the hedonic liking of the bread sample using a 7-point hedonic scale (“1—dislike very much” to “7—like very much”). Than the perception of the saltiness intensity of the bread samples was assessed using a 5-point just about right (JAR) scale, with saltiness levels as follows: “1—way too little”, “2—too little”, “3—just about right”, “4—too much”, “5—way too much”.

Between the sensory sessions, the participants responded to a set of behavioural and attitudinal questions related to their bread consumption and preferences (frequency of bread consumption, preferences for bread type, importance of various intrinsic and extrinsic attributes of bread, etc.) which was done primarily for further consumer segmentation, but also in order to mitigate the panellists’ sensory fatigue. After the completion of the second session of sensory evaluation, the final set of questions related to various aspects of their salt consumption (salt intake, intensity of at-the-table salt addition, status of salt intake control, etc.), and respondents’ socio-demography (age, gender, education and income level). On average, consumers completed the sensory experiment and the questionnaire in forty minutes. After completion, they received a small compensation (a pen and a notebook) for their participation.

### 2.3. Statistical Analyses

#### 2.3.1. Hedonic Liking and Perception of Saltiness Intensity

For the assessment of the existence of significant differences in overall hedonic liking scores (7-point hedonic scale) and perception of the saltiness intensity (5-point JAR scale) between the bread samples, a two-way ANOVA with least significant difference (LSD) multiple comparisons post-hoc test was used. Differences were considered significant at *p* < 0.05. In order to identify the factors that may further explain the consumer sensory responses to the reduction of saltiness in various breads, penalty analysis was applied [48]. This method, also called mean drop analysis, is frequently applied in new product development processes for identifying the directions of product reformulation. Routinely, the test is used to relate a change (usually a decrease) in product liking to individual attributes, allowing for the determination of whether and how intensively the liking scores correlate with these attributes. In our application, the only analysed sensory attribute was saltiness and the association between perceptions of saltiness intensity below optimal (i.e., non-JAR scores) and weaker hedonic liking scores was assessed. First, the JAR scores from the 5-point JAR scale were aggregated into three classes, namely: “not enough salt”—NES (scores 1 and 2), “just about right”—JAR (score 3), and “too much salt” TMS (scores 4 and 5). The *p*-values corresponded to the comparison test of the mean for the JAR scores and the means for the two non-JAR scores were evaluated. XLSTAT software [49] was used to perform the penalty analysis.

#### 2.3.2. Consumer Segmentation

To model for sample heterogeneity, one of the available analytical methods for consumer segmentation was applied, namely the latent class cluster analysis (LCA). LCA enables a useful division of a population into a number of classes which best fit the data, assigning a probability of each variable to each class [50] and a class membership prediction to each sample as well as calculating the predicted modal posterior membership probabilities. In order to determine the best underlying model, goodness-of-fit measures such as log-likelihood, likelihood-ratio chi-square (G2) and parsimony statistics such as the Bayesian information criterion (BIC) [51] and the Akaike information criterion (AIC) [52] were used in this study. To perform the LCA, the poLCA package for the R software environment [53] was used. Since the number of latent variables was unknown, the analysis was repeated for a number of classes, starting with 2, until the best values for the Bayesian information criterion was achieved. The respondents were divided into clusters according to their scores of perceived saltiness intensity level. Descriptive socio-demographic characteristics are analysed and presented for all respondents and per different segments.

#### 2.3.3. Principal Component Analysis

Principal component analysis (PCA) was used to determine the underlying factors explaining the patterns of correlation within the analysed set of bread attributes’ degree of importance in purchasing behaviour. The aim was to identify the key factors that explain the largest proportion of the variance observed in the importance assigned to bread attributes. Here Kaiser’s criterion or the eigenvalue rule and the Scree test were used to determine the number of components. Finally, the derived utility components of the total sample were mapped against the position of the three segments derived in the LCA analysis across the cognitive utility dimensions. Here we explored associations between consumer heterogeneity in terms of their perceived saltiness intensity level with the underlying bread-related behavioural determinants. The software STATA, version 15.1 [54], was used to perform the PCA.

## 3. Results

### 3.1. Hedonic Liking and Perception of Saltiness Intensity

Salt concentration did not have a significant influence on consumer overall liking score for the white bread (WB) samples, whereas the hedonic liking score for the multigrain breads (MGB) was affected significantly. As shown in Table 2, the hedonic liking score for the reference MGB2 (1.3 g salt/100 g) significantly differed from both the low salt MGB1 (1.1 g/100 g) and high salt MGB3 (1.4 g/100 g). The liking scores for MGB samples form an expected inverted U-shaped curve, with MGB2 receiving the highest hedonic liking, whereas the low salt sample (MGB1) was scored lowest. Therefore, for multigrain breads, salt reduction might result in some negative consequences on consumer liking, whereas for white breads, salt reduction of at least 15 percent could be done without risking a negative consumer response.

Furthermore, results confirm a rather low ability on average of consumers to detect saltiness differences measured with the JAR scale. Firstly, no significant differences were observed in the perception of saltiness intensity level between the samples. On average, all breads were scored slightly below JAR, hence perceived as slightly unsalted. Furthermore, the JAR scores and salt concentration in breads are related against expectations, which is mostly, but not statistically significantly evident for the MGB samples. The respondents scored the bread with highest salt concentration (MGB3) as the least salty.

Finally, the results from the two-way ANOVA and least significant difference reconfirm the importance of the bread matrix, since the type of bread was a significant factor of hedonic liking score (*p* < 0.001), with the WB samples receiving higher scores, whereas the salt concentration was not significant (*p* = 0.135). The interaction between the type of bread and salt concentration was significantly influencing the overall bread liking score (*p* = 0.055). As far as the effects of the two dependent variables on the perception of saltiness intensity measured with the JAR scale, no significant interaction was found (Table 2).

In order to further investigate consumers’ reactions towards the samples with perceived non-optimal saltiness levels, penalty analysis was performed. The results presented in Table 3 show that all but one mean drop (MGB1), which is a difference between the mean liking score of consumers perceiving the sample as JAR and of the two sub groups perceiving the bread as TMS or NES, are significant at *p* < 0.01. The scores for the perceived saltiness intensity were distributed comparably for all the bread samples. Between 60% and 65% of the consumers expressed that the saltiness level of the breads is appropriate. A notable exception here is the MGB1 sample, where (only) half of the consumers perceived the saltiness level as JAR, whereas 29% perceived it as NES and 21% as TMS. It is clear that hedonic liking scores systematically decreased due to the perceived non-optimal saltiness level. According to the total penalty score, the bread sample where the consumers most intensively penalised non-optimal saltiness level is WB3, followed by MGB2. This is against expectations, since the former sample is the white bread with highest salt concentration, and the latter is formulated according to the most popular multigrain bread on the Slovenian market. However, these results show notable consumer heterogeneity, and most importantly, a significant degree of penalisation by the “non-JAR” sub-groups of the respondents.

**Figure 1:**
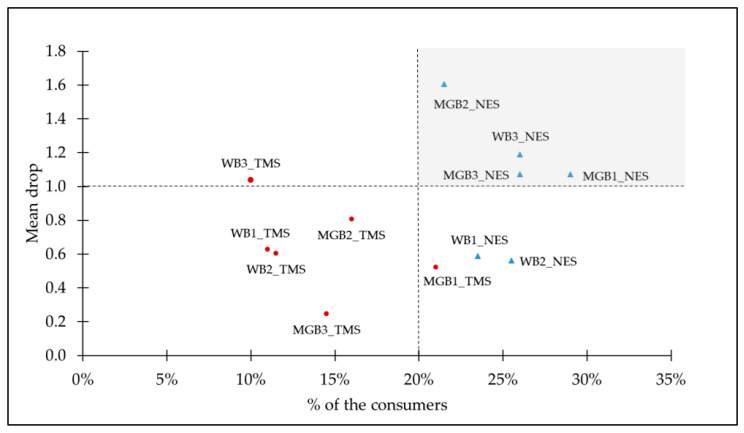
Penalty analysis. Mean liking drops in points on a 7-point hedonic scale and size of the non-JAR sub-groups (%) (*n* = 200).

Further insight of the magnitude of the “non-JAR” sub-groups hedonic liking penalisation is provided in the Figure 1. All three MGB samples and the WB3 sample are located in the so-called critical corner of penalty analysis (>20% of consumers and >1.0-point mean liking drop) [55] and for the critical corner cases, the samples were perceived as “not enough salt”. The most significant mean liking score drop was revealed for the MGB2 sample by the NES sub-group, where 21% of the consumers on average assigned a 1.604 points lower hedonic liking to the sample compared to the JAR sub-group (*p* < 0.0001). Clearly, the perceived sub-optimal saltiness level significantly affects hedonic liking, particularly for multigrain breads.

### 3.2. Segmentation Based on the Perception of Saltiness Intensity in Bread Samples

In order to further explore the revealed consumer heterogeneity related to the perception of saltiness level in breads and a significant degree of penalisation in cases of perceived sub-optimal saltiness intensity, a segmentation using the latent class cluster analysis was sought. Our goal here was to relate the perception of saltiness intensity scores with socio-demographic attributes, bread consumption behaviour and attitudes and behaviours related to salt consumption. As is evident from Table 4, the highest goodness-of-fit measures were obtained from the three-cluster solution when dividing the subjects according to their perception of the saltiness intensity of breads. Relevant measures were used to determine the optimal number of clusters, namely the log-likelihood criteria in conjunction with the comparison of classification error and number of parameters [51,56]. By comparing models with different numbers of latent classes, a model was selected with the fewest number of parameters and the lowest BIC_LL_ before it starts rising again. Bayesian information criteria on the log-likelihood (BIC_LL_) is one of the parameters commonly used to select the optimal number of latent classes in a model.

Based on consumers’ reactions towards the salt reduction in bread, the latent class cluster analysis yielded three distinct groups of consumers with a significant and meaningful heterogeneity. Wald statistics showed that all indicators differ significantly between the classes. The *R*^2^ values reveal that the variance of each indicator explained by the three-class model ranges from 26% to 36%.

Item mean scores for perception of saltiness intensity in various bread samples of the three identified clusters, together with the cluster sizes, are presented in Table 5. The segment loadings found on each cluster were strong with no cross-loadings, and all three indicators significantly differ between the clusters. Roughly two-thirds of the subjects (69.3%) are classified into the group named “salt indifferent”, followed by “salt adherent” subjects (21.6%), whereas the third sub-group includes “salt sensitive” consumers (9.1%). The three segments were labelled and interpreted on the basis of the average JAR score of the sub-group. Therefore, the “salt adherent” cluster members consistently rated all the bread samples as having not enough salt, and reversely the “salt sensitive” cluster members perceived the samples as having too much salt.

Socio-demographic characteristics of the three clusters are presented in the Table 6. Using a *z*-test with Bonferroni adjustment for the difference between proportions, significant differences were found for some levels in age and employment status, whereas for different levels in gender, education and financial status, no differences were detected. As far as age is concerned, the “salt sensitive” cluster shows a bipolar age distribution, whereas the “salt indifferent” and “salt adherent” clusters tend to be biased towards the younger age groups. The average age of the “salt sensitive” cluster is 47.2 years, whereas for the “salt indifferent” and the “salt adherent” clusters, the average age is 39.0 years and 38.9 years, respectively. Despite the fact that gender is not significantly different among the clusters, there is a noteworthy observation where, especially for the “salt sensitive” segment, the proportion of females is notably higher than on average. The “salt adherent” cluster has a notably higher share of members, with at least a university level of education, and the “salt sensitive” cluster has much lower proportion of consumers with an above-average financial status.

The comparison of bread purchasing behaviour and consumption-related characteristics of the total sample and the three clusters is presented in Table 7. There is no difference in the frequency of bread consumption and the place of purchase, but a notably higher share of the “salt adherent” cluster consume bread a few times a day compared to the “salt sensitive” cluster. Moreover, a somewhat higher share of the “salt sensitive” cluster members buy bread from hard discount stores, while the majority of consumers still buy bread from traditional retail chains. On average, more than half of the consumers in the whole sample classify themselves as extendedly loyal buyers of breads, stating that they always choose their purchase from a narrow and constant selection of breads. Similarly, for the three clusters, the majority of members classify themselves as extendedly loyal buyers, but there is a significant difference between the clusters in the size of the consumers classified as variety seekers. The “salt adherent” cluster has less than one-tenth of the members in the variety seeker category, whereas more than one-third of the “salt sensitive” cluster state that they like to choose and try different breads. Only an insignificantly lower share of variety seekers is revealed in the “salt indifferent” cluster. The multigrain bread group received the highest share when consumers were asked to indicate their preferred type of bread. More than one-third of consumers choose breads in this category most frequently, which in Slovenia includes breads based on wheat flour and mainly rye flour, but buckwheat, spelt and corn breads are also getting popular. Wheat breads made from wholemeal or refined flour are next in line, as the former is the most frequently consumed bread for almost 26% of consumers, while white bread consumers account for one-fifth of the sample. Finally, multigrain breads with seed toppings represent the remaining preference for one-fifth of consumers. The differences between the clusters certainly lie in the preference for wholemeal white bread, as half of the “salt sensitive” cluster chose it as their preference, compared to only approximately one-fifth of the “salt adherent” cluster; their preference is for white bread, but only one-tenth of “salt sensitive” consumers chose this bread. The origin of flour and the producer are the two most important extrinsic attributes when the consumers are selecting bread, and both received the same average importance score from the whole sample. The price level of bread is scored just below the mid-point of the importance scale, while the promotional pricing and advertising of bread are perceived as the least important determinants of the purchasing decision. When the scores of the three clusters are compared, there is no significant difference in mean scores and the ranking of the importance is the same, however the overall lower importance rating given by the “salt sensitive” cluster for all extrinsic attributes is noteworthy. Similarly, lower importance ratings by the “salt sensitive” cluster are also evident for the intrinsic attributes of bread. The most important intrinsic factor for all clusters when selecting bread is taste, but the “salt sensitive” group rates the importance of this factor significantly lower (*p* < 0.05) compared to the “salt adherent” cluster. For the remaining intrinsic quality attributes, there are no notable differences and all the determinants are rated above the mid-point of the five-level importance scale. Finally, the least important intrinsic attribute of bread for the whole sample, including the “salt adherent” and “salt indifferent” clusters, is the salt content, however the “salt sensitive” cluster rates this attribute as slightly more important than the high fibre content and no additives in bread attributes.

### 3.3. Principal Component Analysis on Perceived Importance of Extrinsic and Intrinsic Attributes of Bread

**Figure 2:**
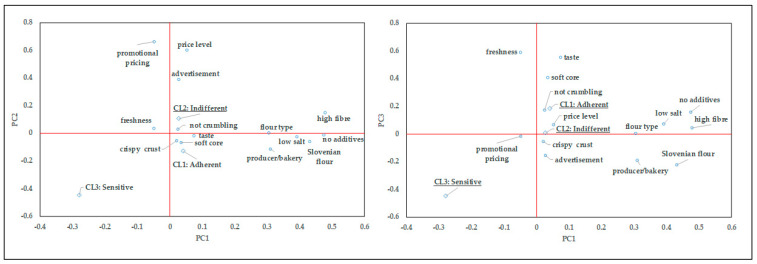
Loading plots of the principal component analysis for the variables related to the importance of extrinsic and intrinsic attributes in bread selection (*n* = 200).

A principal component analysis (PCA) was used in order to generate a simplified view of the difference between the clusters on the importance of extrinsic and intrinsic attributes of bread and to extrapolate the main sources of variation. First, the data set was reduced to a smaller set of underlying factors based on the correlations of the original variables; Table 8 shows the results of the Eigen analysis of the correlation matrix. The cross-validation technique revealed that the PCA can only partially explain the total variability, since the first principal component explained 19.4% of the total variation, 32.5% was explained by the first two components and 44.1% by first three. Although the performed linear transformation employing orthogonal Varimax rotation and Kaiser normalisation was not highly efficient, the newly generated dimensional space was useful to better visualise the relevant variables and positions of the clusters. The PCA loading plots in the plane of the three principal components are presented in Figure 2.

The variables that are most closely correlated with the first component are mainly intrinsic, such as high fibre content, no additives, low salt content and Slovenian origin of the bread ingredients. The first three variables in this group are somewhat health-related, and PC1 effectively separates them from the sensory and rheological variables (e.g., taste, not crumbling, soft core, crispy crust), which, however, have a rather low correlation with PC1. It is shown that the “salt adherent” cluster is clearly associated with this group of variables. The solitary position of the “salt sensitive” cluster is surprisingly defined by an indicative negative correlation to the health-related variables, including the low salt content in bread. Similarly, a remarkable distance can be observed on the PC2 axis, where marketing related variables are grouped. The “salt sensitive” cluster appears to be composed of self-confident individuals who somehow have a negative opinion, which is also confirmed by the PC3 axis. This dimension also clearly separates the health-related variables in a group. The “salt adherent” cluster faces the sensory and rheological variables, while the importance of price in bread selection is reduced by this dimension. The results of the PCA confirm a remarkable heterogeneity of consumers also in terms of the importance they attach to different extrinsic and intrinsic attributes in bread selection, and further reveal notable characteristics of the cluster members.

### 3.4. Salt Use-Related Behaviour and Attitudes

As well as salt concentration in processed foods, salt use while preparing or consuming dishes affects overall salt intake and leads to further complexity in disentangling the salt-related public health challenges. Table 9 shows various behaviour and attitude variables related to salt use. Significant differences were found, and in particular, it is worth noting the differences with regard to salt adding practices. Already the variations in the frequencies of adding salt to various dishes by the clusters show a clear distinction in behaviour. At the aggregate level, consumers from the “salt adherent” cluster expectedly add salt to all of the evaluated dishes notably more frequently. The clear distinction continued also when consumers were asked to evaluate their salt intake. The opinion of the “salt adherent” cluster subjects is statistically significantly higher towards the awareness of their excessive salt consumption. Furthermore, the “salt adherent” cluster members are significantly less attentive toward salt consumption. Only 5% of this cluster’s members are always attentive to salt intake in contrast to the “salt sensitive” cluster (35%) and even to the “salt indifferent” subjects (17%). On average, 16% of the total sample is very attentive to salt intake and a further 56% occasionally pay attention. The proportion of subjects in the whole sample that do not pay attention to salt intake is 29%. The awareness of the effects of excessive salt consumption on health is quite high and homogenous between the clusters. Finally, the largest share of the consumers in the sample believe that the most appropriate approach for managing the salt intake is careful use of salt during cooking (36%), followed by the limitation of salt addition at the table (32%). Here, significant differences are observed between the clusters, where the subjects from the “salt adherent” sub-group show a significantly lower level of agreement that the limitation of adding salt is the optimal strategy for salt consumption reduction.

## 4. Discussion

In contemporary, highly competitive markets, bakers and other food manufacturers perceive salt reduction as highly risky activity due to concerns that the reformulated foods will not meet the expected sensory characteristics enjoyed by the consumers, which would result in a loss of sales. This is despite the fact that several academic studies showed the opposite—that consumers are rather insensitive to small salt reductions [32,33,57,58,59]. The present study is therefore conceptualized on industrial-driven interest with a relatively small (but highly relevant) level of modification in the salt content, using the market-leading bread formulations as a reference. This study confirmed a very low sensitivity to differences in the tested saltiness levels, however several interesting insights were revealed.

The first important observation is related to the impact of the bread matrix on the consumer response to modifications in salt content. As shown in our research, the tested differences in salt concentration did not have a significant influence on the consumer hedonic liking score for white breads, whereas the hedonic liking score for multigrain type breads was statistically significantly reduced in the sample with a 15% lower salt level (1.1 g/100 g). This means that for multigrain breads, the salt level reduction in line with the recommendation of the national plan on salt reduction [42] to 1.0 g/100 g is highly likely to be noticed by the consumers, if not accompanied by other technological measures.

Although salt is a minor component in bread formulations, it has a critical and complex effect on the bread dough system [23,60]. Salt toughens gluten and make dough more stable. It also affects the rate of fermentation, reduces the rate of gas production, and thus significantly influences the bread making process. However, from the perspective of our study, one should not underestimate the effect of salt on other organoleptic properties, besides (salty) taste. Namely, salt has also an important impact on bread texture (mouth feel), overall flavour and colour [21,22,23,46,61].

Therefore, food manufacturers should consider not only a gradual salt reduction approach, but also the use of salt substitutes, flavour enhancers and novel technologies as accompanying measures to salt reduction [15,62,63]. Our results reinforce an important but often overlooked fact that saltiness perception depends on the food matrix in which salt is added [61,64]. To the best of our knowledge, not many other studies on salt reduction in bread point out this important matter. An older study by Wyatt et al. [65] for example reported that the salt content of white or whole wheat bread could be reduced by 50% without any change in flavour and overall acceptability. Most other studies consider bread in a rather generic manner. For example, a meta-analysis of eight studies [58] showed that a reduction in plain bread salt content of more than 40% significantly reduced acceptability. Further, studies suggest that salt content in bread could be reduced by up to 30% without affecting its liking score among consumers [27,29,30,32,33,34,58]. Also, the comparison of consumer sensory and hedonic perception of salt-reduced breads in a two-bite evaluation and (classic) single-bite evaluation did not find significant differences. However, the overall liking scores obtained from the second bite evaluation better reflected differences among samples according to their actual saltiness level [66].

Bolhuis et al. [59] showed that with the use of different sandwich fillings, it is possible to reduce salt level in brown bread by up to 52% without the use of substitutes or sodium intake compensation by choice of filings; also, no lowering of food consumption was observed. However, the study by Rødbotten et al. [67] working with barley bread with normal and reduced (−50%) salt content (starting at 1.3% of salt by weight of flour) reported a significant decrease in consumer liking score. It is evident from our results that approaches for salt reduction should be made on a product-specific basis even within the same food category. Therefore, for multigrain breads, salt reduction must be implemented more carefully, whereas for white breads, salt reduction of at least 15% can be done without risking a negative consumer response.

To provide further insights, our next focus was the analysis of hedonic liking vs. the perceived intensity of the saltiness level. When consumers were asked to assess the saltiness intensity of the bread samples using the JAR scale, aggregated analysis yielded no significant differences. The consumers, on average, perceived all the bread samples in our study as slightly unsalted—i.e., they scored the samples slightly below the JAR salty level. Moreover, when both the perceived saltiness intensity scores and actual salt concentration in breads are correlated, inconsistent patterns are revealed, suggesting that untrained consumers are unable to discriminate modification of the salt levels in testing breads. For example, the respondents scored the multigrain bread (MGB3) with highest salt level (1.4 g/100 g) as the least intense in saltiness, whereas for all white bread samples, the perceived intensity of saltiness is practically identical. Therefore, modification of the salt content did not evoke differences in saltiness intensity perception. However, previous researchers warn that separately considering the consumer’s hedonic liking of a product and a sensory attribute intensity perception (e.g., saltiness) can result in misleading conclusions [68,69]. In order to diminish this risk, our research combined hedonic liking and JAR scores using the penalty analysis, which provided further valuable information. The results show that consumers penalize hedonic liking scores if breads are perceived as non-optimally salty. The two sub-groups of consumers perceiving the bread as having either “too much salt” or “not enough salt” gave statistically significant lower liking scores in comparison to the “JAR” sub-group. Therefore, perceived saltiness intensity was a clear liking driver for hedonic liking scores, which systematically decreased due to the perceived non-optimal saltiness level. The already-described relationship between liking and saltiness intensity [70] has been confirmed, since the results form an inverted U-shaped curve with highest hedonic liking for the breads with an optimum salt intensity (JAR salty level) and overall liking decreases above and below this point. Therefore, in order to successfully reformulate “less healthy” foods, producers should understand complex relations between sensory attributes and consumer liking [15]. In the case of salt reduction in breads, the objective is to avoid diminishing the perceived intensity of the key liking drivers, and salt plays an important role in bread despite the fact that saltiness itself is not a dominant flavour of this product category. It is responsible for overall flavour profile, amplifying sweetness, and masking bitterness [22,71].

Additionally, results of the penalty analysis revealed a significant degree of heterogeneity of the consumers regarding their response towards perceived differences in saltiness intensity. This reinforces the somehow overlooked challenge for food producers that it is not necessarily appropriate to treat all consumers with a single strategy for salt reduction. The number of studies recognising the importance of considering potential differences between consumers in food product reformulation success has only recently increased [26,57,72,73,74]. In order to contribute towards the understanding of this aspect of food reformulation, the present research provides further confirmation of previously reported differences between consumers. The segmentation is based on the perceived saltiness intensity measured with the JAR scale, since the correlation of product liking and perceived saltiness intensity level has been clearly confirmed [75,76,77] but not extensively studied. Latent class clustering identified three clusters differing significantly in the saltiness perception of breads. The “salt adherent” consumers consistently perceived bread samples as having not enough salt; they tend to be more common in the lower–mid age groups of the population and are rarely retired. They also report a higher education level and are more commonly male. Vice versa, the “salt sensitive” cluster members are predominantly female, and more often either retired or students, which gives the cluster a bipolar age distribution. The perceived saltiness intensity of the “salt sensitive” cluster was higher for all breads. Approximately two-thirds of consumers fall into the “salt indifferent” cluster, and their perceived saltiness was in between the two abovementioned clusters. Since the hedonic liking of the non-optimal saltiness level significantly affects hedonic liking score, it is important to focus on the consumers perceiving breads as not-JAR for salt and to understand their bread purchasing behaviours and consumption-related characteristics. This is due to the reduced risk of adverse effects from bread reformulation, which is an important barrier for industrial activities in salt reduction programs.

Our results also show that the “salt sensitive” cluster systematically assign lower importance rates for the extrinsic attributes of breads compared to the other two clusters; however, the rankings of attributes are very similar. A similar pattern was also revealed for the intrinsic bread attributes, but it is noteworthy to point out two explicit differences. The “salt sensitive” group ranks the taste of bread as the most important attribute, but importance is significantly lower than for the other two clusters. Finally, the least important intrinsic attribute of bread for the “salt adherent” and “salt indifferent” clusters is the salt content, however the “salt sensitive” cluster rates this attribute as slightly more important than the high fibre content and no additives in bread attributes. This is in contrast with the conclusions of other researchers who underlined that the perceived individual health benefits present an essential motivation in bread choice [78,79]. The low importance of health-related bread attributes (e.g., low salt, high fibre and no additives) is also confirmed by the result of the PCA. When the main sources of variation were extrapolated, one can conclude that the “salt adherent” cluster is clearly associated with the sensory and rheological variables of bread (e.g., taste, not crumbling, soft core, crispy crust), whereas the “salt sensitive” cluster stepped out by an indicative negative correlation to the health-related variables, and similar trends were revealed for intrinsic attributes and the marketing-related variables. It is clear that the so called “credence quality attributes” [80], which are not self-evident and are based on consumer trust, have relatively limited importance when purchasing bread. Consumer quality perception of bread is mainly determined by sensory and health attributes, which further influence purchasing decision making [81]. Such complexity might be assigned to various determinants, however consumers’ perceived relevance of determinants for their own food choices are not necessarily the most important explaining factors [82,83]. Habits related to eating and acquiring foods explain a large number of choices, which is also of particular importance when the saltiness in food is concerned [84], followed by food experiences by culture, age, gender and taste phenotype [75,77,85]. Certainly, the process of bread reformulation should be managed by considering the related eating habits and individual differences in this respect. Our results also confirm significant variability, particularly with regard to practices and behaviours related to adding salt by consumers. Furthermore, previous research shows that people with high-sodium diets tend to prefer foods with higher sodium levels [86,87], whereas the opposite trend has been observed for those who follow low-sodium diets [86,88,89]. Our results show that the “salt adherent” cluster members are notably heavier in habit of adding additional salt at the table, but they are also highly aware of their excessive salt use and significantly less attentive in limiting their salt consumption. Studies suggest that general changes in dietary habits are needed for an effective decrease in salt content to affect NCD risks [90,91,92]. A survey by Mørk et al. [74] showed that consumers who have already changed their dietary salt intake or intend to do so are also more willing to purchase salt-reduced food products. To change dietary habits, salt awareness and knowledge needs to be increased, since significant deficits have been detected in this respect. Sarmugam and Worsley [93] reported that consumers recognize health risks associated with high salt intake, but knowledge of recommended daily intakes, understanding of the salt–sodium ratio, and foods that contribute the most salt to diet is poor. On the other hand, our results showed quite high and homogenous awareness of the negative health consequences from excessive salt consumption. This is comparable with the study by Newson et al. [25] that also showed high level of consumer awareness in this respect and understanding the necessity of salt intake control. Also, Di Vita et al. [94] showed evidence of a certain risk consciousness among the more vulnerable subjects, such as overweight individuals. Moreover, they found that body mass index (BMI) significantly influences willingness to pay (WTP) for low salt bread alternatives, highlighting that this specific sub-group of consumers shows positive intention to pay a price premium for healthier alternatives. Certainly, specific groups of consumers are increasingly oriented towards healthier diets and consequently bread producers are extending their assortment with healthier options such as organic, whole grain, ancient grain (pseudo-cereals) or sourdough breads. Health benefits are often among essential motivations for the choice of bread, but research shows that consumers are not necessarily aware of their main salt intake source [25,31]. When they are asked to estimate where the most salt in their diet comes from, 43% of consumers globally reported that their main salt intake source was salt added during cooking [25]. Our results also suggest that the consumers are usually not aware of the main sources of salt intake. The largest share of the consumers in our research believe that the most appropriate approach for managing salt intake is careful use of salt during cooking (36%), followed by the limitation of the addition of salt at the table (32%). This is despite the commonly agreed fact that in developed countries, only about 15% of dietary salt originates from addition during cooking or at the table [11,16]. Furthermore, non-adherence to salt restriction appears to be a complex problem and studies showed that consumer behaviour change based on education and awareness-raising interventions alone are unlikely to be adequate in reducing population salt intake to the recommended levels [95]. A significant difference was observed between the clusters in this study, where the “salt sensitive” subjects highly agree that limiting the use of salt at the table is the most appropriate approach to reduce salt consumption in the population, and the level of agreement from the “salt adherent” cluster is statistically significantly lower. Finally, the full sample and all the clusters place the selection of breads with lower salt content as the least effective approach to limit salt intake, which brings out the issue of consumer information deficit. Adequate consumer understanding and awareness related to the sources of dietary salt and adverse health consequences of excessive salt consumption is a precondition for necessary habit changes, and in combination with optimal management of other closely related processes (e.g., scientific understanding, industrial reformulation and government incentives), will lead to the nutritional optimization of processed foods [15]. Evidence of the positive effects of food product reformulation are strong for sodium interventions [17], which means that reducing salt in bread as one of the main salt sources in our diet may be one of the most effective strategies to reduce the risk of NCDs. The best achievements in salt reduction can be achieved with a combination of multiple population-wide regulatory policies that also include mandatory reformulation and food labelling [96]. It should be also mentioned that sensory acceptability of low-salt foods can be also achieved with the use of herbs and other no-salt seasoning blends [97], but is likely that such foods will be of particular interest to more health-conscious consumers, while a reduction of salt in the market-leading types of bread will affect much larger population groups.

A major strength of this study is the inclusion of two types of breads, both of which were based on market-leading bread formulations taken as a reference for the experimental samples. The consumer sensory evaluation was done on a relatively large sample, making the results particularly relevant. While non-probability sampling was used, we aimed for a balanced representation of genders and age groups. A possible limitation of the study could be that we tested samples with relatively small differences in salt levels (national average; −15%; +10%), which turned out to be challenging for consumers to detect. However, this approach was intentional and can be also considered as study strength. Namely, the tested levels of salt concentration are highly pertinent with regard to the real reformulation targets of Slovenia and many other countries, as well as currently existing levels of salt in breads on the market. While we would probably be able to detect more significant consumer responses if larger differences in salt level were applied, the results of such a study would be less relevant for meeting real-life food reformulation challenges. Bread is also rarely consumed alone, thus our study design could be upgraded by the addition of commonly eaten spreads or sandwich fillings [59], and may as such give more relevant, real-life results. A further limitation of the study is also the focus on overall liking and saltines perception in breads, whereas reduction of salt clearly also influences other bread quality characteristics which are important for consumer acceptance. Therefore, further research is needed to analyse the effects of salt reduction on other flavour constituents, particularly for multigrain breads. Better insight is needed for clear recommendations to the baking industry on how to retain the sensory characteristics of the salt-reduced multigrain breads. Finally, another possible limitation of the study could be that we did not explicitly evaluate some relevant physiological parameters (e.g., hunger, thirst) of the consumers which could affect sensory evaluation and saltiness level perception [98,99]. This would certainly improve the quality of the results.

## 5. Conclusions

Our results show that in breads, the food matrix should be considered when studying the hedonic liking and saltiness perception in the context of salt reduction. Consumer hedonic liking was not affected by 15% salt reduction for white breads, whereas for multigrain breads consumers showed sensitivity and significantly reduced liking score. Tested modification in the salt level in bread samples did not evoke differences in consumers’ saltiness intensity perception, however when scores of perceived saltiness intensity were considered along with hedonic scores, results revealed that consumers perceiving bread samples as non-optimally salty significantly penalised hedonic liking scores. Perceived saltiness was a clear liking driver for hedonic liking scores and the underlying consumer heterogeneity was found to be related to the importance of extrinsic and intrinsic attributes of bread, as well as habits related to salt use. Segmentation based on perceived saltiness identified three clusters (salt adherent, salt indifferent, and salt sensitive consumers), but the importance of various health related attributes of bread for bread purchase was found to be low in all three, despite a common awareness of adverse effects of excessive salt consumption on health. Moreover, our results suggest that the consumers were not aware of the main sources of salt in their diets, nor did they recognise consumption of bread with reduced salt content as an important strategy for limiting dietary salt intake. Still, the three clusters of consumers showed significant variability in practices and behaviour related to salt-adding.

It is clear that public incentives for industry-led reformulation of breads (and other products) is a highly promising approach for reducing dietary salt intake, but the importance of the bread matrix and consumer heterogeneity should be considered in designing the public health or industry level activities. Baking industry should pay attention to the consumers who perceive bread saltiness as non-optimal, their dietary habits and bread-purchasing behaviour in terms of product differentiation and communication of healthy aspects of reformulated products. For some of the producers this might improve their market position and competitiveness. In addition, our research supports further government stimulated information activities that may improve the level of consumers’ nutrition-related health literacy with regard to food reformulation and consequently increase the motivation for diet modification towards healthier food choice and behaviour. However, these public interventions should also be better targeted and individual differences should be considered which is an important implication for policy makers. Only strategically coordinated multi-actor approach could ameliorate the negative health impacts of excessive global salt consumption.

## Figures and Tables

**Table 1:** Bread sample formulations.

Sample	Salt Content (g/100 g of Bread)	Ingredients (% of Flour Weight)					
		Wheat Flour (500 Type)	Rye Flour (1500 Type)	Water	Yeast	Improver	Salt
WB1	1.1	100%	/	60%	2.5%	2.5%	1.58%
WB2	1.3	100%	/	60%	2.5%	2.5%	2.87%
WB3	1.4	100%	/	60%	2.5%	2.5%	2.02%
MGB1	1.1	70%	30%	60%	2.5%	2.5%	1.65%
MGB2	1.3	70%	30%	60%	2.5%	2.5%	1.98%
MGB3	1.4	70%	30%	60%	2.5%	2.5%	2.10%

WB—white bread; MGB—multigrain bread.

**Table 2:** Descriptive analysis of the hedonic liking (7-point hedonic scale) and perception of saltiness intensity (5-point just about right (JAR) scale) of the bread samples with different salt concentrations using two-way analysis of variance (ANOVA) and least significant difference (LSD) (*n* = 200).

Sample	Salt Concentration (g/100 g of Product)	Hedonic Liking Score	JAR Score
		Means (CI)	Means (CI)
WB1	1.1 g	4.79 (4.59–4.98)	2.39 (2.29–2.51)
WB2	1.3 g	4.74 (4.54–4.94)	2.36 (2.26–2.54)
WB3	1.4 g	4.78 (4.58–4.97)	2.35 (2.25–2.55)
MGB1	1.1 g	3.84 (3.64–4.04) ^a^	2.40 (2.30–2.50)
MGB2	1.3 g	4.27 (4.07–4.47) ^b^	2.45 (2.34–2.55)
MGB3	1.4 g	4.14 (3.94–3.34) ^ab^	2.36 (2.26–2.54)

Note: Different superscript letters indicate group means that are different at the 5% significance level. *p*-values of two–way ANOVA: (1) Overall liking score: type of bread *p* < 0.001; saltiness level *p* = 0.135; interaction effect *p* = 0.055. (2) JAR score: type of bread *p* = 0.378; saltiness level *p* = 0.639; interaction effect *p* = 0.712. CI—confidence interval.

**Table 3:** Penalty analysis table with mean overall liking scores (7-point hedonic scale) by JAR and non-JAR subgroups of consumers (5-point JAR scale for saltiness level) (*n* = 200).

Sample	Perceived Saltiness Level ^1^	Consumers (%)	Mean Liking Score	Significance of Mean Drop ^2^ (*p*-Value)	Total Penalty
WB1	*NES*	23.5	4.404	0.011	0.601
	*JAR*	65.5	4.992		
	*TMS*	11.0	4.364		
WB2	*NES*	25.5	4.392	0.011	0.574
	*JAR*	63.0	4.952		
	*TMS*	11.5	4.348		
WB3	*NES*	26.0	4.000	<0.0001	1.146
	*JAR*	64.0	5.188		
	*TMS*	10.0	4.150		
MGB1	*NES*	29.0	3.190	<0.0001	0.840
	*JAR*	50.0	4.260		
	*TMS*	21.0	3.738	0.126	
MGB2	*NES*	21.5	3.140	<0.0001	1.264
	*JAR*	62.5	4.744		
	*TMS*	16.0	3.938		
MGB3	*NES*	26.0	3.385	<0.0001	0.775
	*JAR*	59.5	4.454		
	*TMS*	14.5	4.207		

Note^1^: NES—“not enough salt”; JAR—“just about right”; TMS—“too much salt”. Note^2^: significance of mean drop in overall liking based on 2-sample *t*-test.

**Table 4:** Latent class cluster models’ goodness-of-fit measurements (*n* = 200).

Model	LL	BIC_LL_	AIC_LL_	G2	N (Par)	Class. Err.
One-cluster model	−1257.37	2636.61	2560.75	757.84	23	0
Two-cluster model	−1199.72	2558.40	2459.45	642.54	30	0.100
Three-cluster model	−1171.31	2538.66	2416.63	585.72	37	0.103
Four-cluster model	−1162.32	2557.77	2412.64	567.73	44	0.166

LL—log–likelihood; BIC_LL_—Bayesian information criteria on the log–likelihood; AIC_LL_—Akaike information criterion based on log-likelihood; G2—likelihood-ratio Chi-square; N (Par)—number of classes; Class. Err.—classification error.

**Table 5:** Cluster sizes, class-specific marginal means and parameter estimates for the perception of saltiness intensity in breads (5-point JAR) (*n* = 200).

Bread Sample	Perception of Saltiness Intensity (JAR) by Clusters			Cluster Loading Indicators		
	CL1: Salt Adherent	CL2: Salt Indifferent	CL3: Salt Sensitive	Wald	*p*-Value	*R* ^2^
WB1 (1.1 g)	1.95	2.41	3.33	23.70	0.00	0.32
WB2 (1.3 g)	1.79	2.40	3.44	24.98	0.00	0.36
WB3 (1.4 g)	1.75	2.44	3.10	22.71	0.00	0.26
MGB1 (1.1 g)	1.81	2.47	3.24	19.41	0.00	0.20
MGB2 (1.3 g)	1.88	2.56	2.95	16.94	0.00	0.24
MGB3 (1.4 g)	1.72	2.45	3.29	26.63	0.00	0.31
Cluster size	21.65	69.29	9.06			
(% of total sample)						

CL1—Cluster 1: Salt adherent; CL2—Cluster 2: Salt indifferent; CL1—Cluster 3: Salt sensitive; Wald—Wald statistics; *p*-Value—significance; *R*^2^—squared correlation.

**Table 6:** Socio-demographic characteristics of the sample and the three clusters (*n* = 200).

Variables	Whole Sample	CL1: Salt Adherent	CL2: Salt Indifferent	CL3: Salt Sensitive
Age groups, years (%)				
<25	23.5	18.6	24.8	25.0
26–35	22.0	25.6	20.4	25.0
36–45	19.0	20.9	21.2	0.0
46–55	22.0	25.6	21.2	20.0
56–65	7.0	7.0	6.6	10.0
>65	6.5	2.3 ^a^	5.8 ^ab^	20.0 ^b^
Gender (%)				
Male	38.0	34.9	41.6	20.0
Female	62.0	65.1	58.4	80.0
Education (%)				
Elementary school	6.5	2.3	6.6	15
High school	36.5	34.9	36.5	40
College	15	9.3	18.2	5
University or more	42	53.5	38.7	40
Financial status (%)				
Below average	11.5	18.6	9.5	10
Average	66.5	55.8	67.2	85
Above average	22.0	25.6	23.4	5
Employment status (%)				
Student	21	16.3	21.2	30
Employed	68	76.7 ^a^	68.6 ^ab^	45 ^b^
Unemployed	2	4.7	1.5	0
Retired	9	2.3 ^a^	8.7 ^ab^	25 ^b^

Notes: Each superscript letter denotes a subset of cluster categories whose row proportions do not differ significantly from each other at the 0.05 level using Bonferroni’s method.

**Table 7:** Bread purchasing and consumption-related behaviour of the sample and the three clusters (*n* = 200).

Variables	Whole Sample	CL1: Salt Adherent	CL2: Salt Indifferent	CL3: Salt Sensitive
Bread consumption (%)				
Few times a day	27.5	30.2	27.7	20.0
Once a day	29.0	25.6	29.2	35.0
Almost every day (4–6×/week)	18.5	14.0	19.0	25.0
1–3 times a week	16.0	18.6	16.1	10.0
Once a week	9.0	11.6	8.0	10.0
Purchase location (%)				
Traditional retailers	58.6	62.8	57.7	55.0
Hard discounters	15.5	14.0	14.6	25.0
Bakery store/industrial/	11.5	14.0	10.9	10.0
local bakery/artisanal/	11.5	7.0	14.6	10.0
baking at home	2.0	2.3	2.2	0.0
Variety seeking behaviour for breads (%)				
High loyalty (“If possible, I always buy the same bread.”)	19.2	21.4	18.4	20.0
Extended loyalty (“I choose from a narrow selection of breads.”)	53.5	69.0	50.0	45.0
Variety seeker (“I like to choose and try different breads.”)	27.3	9.5 ^a^	31.6 ^b^	35.0 ^b^
Type of most preferred bread (%)				
Multigrain	35.3	30.0	37.9	27.8
White wholemeal	25.8	22.5	23.5	50.0
White	20.0	27.5	18.9	11.1
Multigrain and seed topping	18.9	20.0	19.7	11.1
Importance of extrinsic attributes of bread (mean; 1–5)				
Origin of flour—Slovenian	3.4	3.5	3.4	3.1
Producer/bakery	3.4	3.5	3.4	3.0
Price level	2.8	2.7	2.9	2.3
Promotional pricing	2.1	2.0	2.2	1.8
Advertisement	1.6	1.5	1.6	1.4
Importance of intrinsic attributes of bread (mean; 1–5)				
Taste	4.7	4.8 ^a^	4.7 ^ab^	4.4 ^b^
Freshness	4.5	4.6	4.5	4.3
Flour type	3.9	4.1	3.9	4.0
Not crumbling	3.6	3.6	3.7	3.5
Soft bread core	3.6	3.8	3.6	3.6
Crispy bread crust	3.6	3.4	3.6	3.7
Without additives	3.5	3.6	3.5	3.3
High fibre content	3.2	3.2	3.3	3.2
Low salt content	3.2	3.0	3.3	3.4

Notes: (1) Each superscript letter denotes a cluster whose row proportions do not differ significantly from each other at the 0.05 level using Bonferroni’s method. (2) Different superscript letters indicate group means that are different at the 5% significance level using Bonferroni’s test.

**Table 8:** Eigen analysis of the correlation matrix using the importance of extrinsic and intrinsic attributes in bread selection (orthogonal Varimax rotation and Kaiser normalisation) (*n* = 200).

Principal Component	Variance	Difference	Variance Explained	
			by PC	Cumulative
PC1	2.72336	0.892466	0.1945	0.1945
PC2	1.83089	0.206025	0.1308	0.3253
PC3	1.62487	0.050120	0.1161	0.4414
PC4	1.57475	0.634294	0.1125	0.5538

**Table 9:** Salt intake-related behaviour of the sample and the three clusters (*n* = 200).

Variables	Whole Sample	CL1: Salt Adherent	CL2: Salt Indifferent	CL3: Salt Sensitive
Habit of adding salt at the table (% of consumers adding salt when eating the dish)				
Eggs	66.0	76.2	68.1	38.5
Salad	57.8	78.3	54.1	41.7
Soup	53.6	65.2	54.0	30.8
Meat	52.8	78.3	52.8	7.7
Bread with dairy spread	11.3	17.4	11.4	0.0
How would you rate your salt intake? (%)				
I believe I use an appropriate amount of salt.	77.0	62.8 ^a^	78.1 ^b^	100 ^1^
I think I use too much salt.	21.0	37.2 ^b^	19.0 ^a^	0 ^1^
I think I use too little salt.	2.0	0.0	2.9	0 ^1^
Are you currently limiting your salt intake? (%)				
Occasionally I pay attention to salt intake.	55.5	60.5	56.2	40.0
No, I do not pay attention to salt intake.	28.5	34.9	27.0	25.0
I am very attentive to salt intake.	16.0	4.7 ^a^	16.8 ^ab^	35.0 ^b^
Do you think too much salt in a diet is harmful? (%)				
Yes, too much salt can be harmful for health.	90.0	90.7	89.1	95.0
I do not know. I am not sure.	9.0	9.3	10.2	0.0
No, too much salt is not harmful for health.	1.0	0.0	0.7	5.0
Which is the most effective approach to limit salt intake in your opinion? (%)				
Careful use of salt during cooking	35.5	37.2	35.8	30.0
Do not add additional salt to cooked dishes	31.5	18.6 ^a^	32.8 ^ab^	50.0 ^b^
Be attentive when eating away from home	11.5	23.3	8.8	5.0
Limit consumption of salty snacks	11.0	11.6	11.7	5.0
Select meat products with a lower salt content	7.0	7.0	7.3	5.0
Select breads with a lower salt content	3.5	2.3	3.6	5.0

Note: Values in the same row and sub-table not sharing the same subscript are significantly different at *p* < 0.05 in the two-sided test of equality for column proportions. Tests assume equal variances. (1) This category is not used in comparisons because its column proportion is equal to zero or one. (2) Tests are adjusted for all pairwise comparisons within a row of each innermost sub-table using the Bonferroni correction.
